# Inherent PD‐L1 22C3 Expression in Alveolar Macrophages Impacts the Combined Positive Score Status in Breast Cancer With Pulmonary Metastasis

**DOI:** 10.1111/1759-7714.70004

**Published:** 2025-03-06

**Authors:** Yoon Jin Cha, Hye Min Kim, Ja Seung Koo

**Affiliations:** ^1^ Department of Pathology Yonsei University College of Medicine Seoul South Korea

**Keywords:** alveolar macrophages, breast cancer, lung metastasis, PD‐L1

## Abstract

**Purpose:**

This study aimed to determine the impact of inherent programmed death‐ligand 1 (PD‐L1)‐expressing alveolar macrophages (AMs) on the combined positive score (CPS) of PD‐L1 (22C3) in metastatic breast cancer in the lungs.

**Methods:**

A total of 87 patients with pulmonary metastases of breast cancer were included in this study. Immunohistochemical staining of various PD‐L1 antibodies was performed. The CPSs and CPSs excluding the number of PD‐L1 positive AMs [CPS(NAM)s] with PD‐L1 (22C3) were determined and compared.

**Results:**

Among 87 enrolled patients, 22 had luminal A breast cancer, 24 had luminal B breast cancer, 13 had HER‐2‐positive breast cancer, and 28 had triple‐negative breast cancer (TNBC). CPSs ≥ 10 was observed only in luminal B (12.5%) and TNBC (35.7%) subtypes (*p* < 0.001), whereas CPS(NAM)s ≥ 10 was observed only in TNBC (14.3%) (*p* = 0.011). Changes from the CPS‐positive to the CPS(NAM)‐negative status occurred in nine cases (10.3%), with significantly higher proportions being observed in the luminal B (12.5%) and TNBC (21.4%) subtypes (*p* = 0.007). Tumors showing changes from the CPS‐positive to the CPS(NAM)‐negative status were larger (*p* < 0.001); similar findings were observed in the TNBC subgroup (*p* = 0.001).

**Conclusion:**

The inclusion of PD‐L1 expressing AMs leads to differences in CPS positivity, especially in large TNBC subtype tumors.

## Graphical abstract

Alveolar macrophages (AMs) in human lung tissues inherently expressed PD‐L1 including 22C3 clone. Therefore, the inclusion or exclusion of AMs significantly influences CPS in lung metastases from breast cancer, particularly in triple negative type.

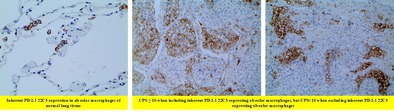

## 1 Introduction

Breast cancer is the most common cancer among women worldwide; one of the most notable reasons for patient mortality is distant metastasis of the tumors. The 5‐year survival rate of patients with metastatic breast cancer is less than 20% [1, 2]. The major sites of metastatic breast cancer are the lungs (21%–32%), bone (30%–60%), liver (15%–32%), and brain (4%–10%) [3]. Approximately 60% of patients with metastatic breast cancer have lung or bone metastases.

The significance of lung metastasis in breast cancer, particularly in triple‐negative breast cancer (TNBC), lies in the following factors: First, high metastasis rates and poor survival outcomes. Lung metastasis is commonly observed in TNBC patients, typically occurring within 5 years of the initial diagnosis. Once lung metastasis develops, it damages normal lung function, leading to symptoms such as coughing, labored breathing, and hemoptysis. These complications ultimately result in death, significantly contributing to morbidity and mortality in breast cancer patients. In fact, 60%–70% of breast cancer patients who succumb to the disease present with lung metastases, and even in cases where lung metastasis is the sole site, the median survival is only 25 months, indicating a grim prognosis [4]. Second, treatment challenges. Chemotherapy, one of the primary treatment modalities for TNBC, has shown limited effectiveness against lung metastases. This is due to poor drug accumulation at metastatic sites, rapid clearance from lung capillaries, and severe adverse effects [5, 6]. Therefore, lung metastasis is a critical determinant of prognosis in TNBC. Early detection and appropriate management of lung metastases are essential for improving patient survival outcomes. In patients with advanced and/or metastatic TNBC, immune checkpoint inhibitors, specifically, pembrolizumab (an anti‐programmed death 1 [PD‐1] antibody), exhibit antitumor effects when used both for monotherapy and in combination with chemotherapy [7, 8, 9].

PD‐1, a PD‐L1 receptor, is expressed on various immune cells as a checkpoint molecule in immune reactions [10]. By binding to PD‐1, PD‐L1‐expressing tumor cells can evade antitumor immune responses [11, 12]. Consequently, determining the PD‐L1 expression status of tumors is crucial for selecting PD‐L1 inhibitors and anticipating treatment responses. The most common and convenient method for investigating PD‐L1 expression is the immunohistochemistry (IHC) analysis of formalin‐fixed paraffin‐embedded (FFPE) tissues using monoclonal anti‐PD‐L1 antibodies. Commercially available monoclonal anti‐PD‐L1 antibodies include those specific for clone 28–8 [13], clone 22C3 [14], clone SP142 [15, 16], and clone E1L3N [17, 18]. Among these, clone 22C3 antibody is the current FDA‐approved diagnostic companion test for treatment with pembrolizumab in TNBC patients [19], based on the result of KEYNOTE‐355 trial, which demonstrated that in patients with PD‐L1‐positive metastatic TNBC, a combination of pembrolizumab and chemotherapy improved progression‐free survival by 4.1 months, extended overall survival to 23 months, and reduced the risk of death by 27%. Evaluation of PD‐L1 (22C3) expression is performed using the combined positive score (CPS), which is the number of PD‐L1‐stained cells (tumor cells, lymphocytes, and macrophages) divided by the total number of viable tumor cells, multiplied by 100. A CPS of ≥ 10, indicating a positive PD‐L1 status, recommends treatment with pembrolizumab.

When a tumor metastasizes to the lungs, tumor cells can reprogram immune cells to increase the expression of immunosuppressive cytokines or immune checkpoint receptors. Therefore, when TNBC lung metastasis is suspected and confirmed through tissue examination, PD‐L1 immunohistochemistry (IHC) should be performed on the lung tissue to measure the CPS in the metastasis. This increases the likelihood of detecting PD‐L1 positivity and provides additional treatment options for immune checkpoint inhibitor therapy. As such, PD‐L1 CPS plays a significant role in managing TNBC with lung metastases.

Alveolar macrophages (AMs) in the lungs inherently express PD‐L1 [20]; therefore, their presence may influence the CPS calculation for TNBC patients with pulmonary metastases. However, the impact of inherent PD‐L1 (22C3) positive AMs on CPS and the mechanisms underlying their impact remain unknown. Therefore, the purpose of this study was to investigate the impact of inherent PD‐L1 positive alveolar macrophages on CPS in breast cancer lung metastases, with the primary goal of determining whether differences in CPS are observed.

## 2 Materials and Methods

### 2.1 Patient Selection and Clinicopathologic Evaluation

We utilized FFPE tissue samples that were obtained from patients diagnosed with metastatic breast cancer in the lungs who underwent excision surgery (such as lung wedge resection) at the Severance Hospital between January 2005 and December 2021. All cases were retrospectively reviewed by a breast pathologist (Koo JS), and histological analysis was performed using hematoxylin and eosin (H&E)‐stained slides.

### 2.2 IHC Analyses

The antibodies used for the IHC analyses are listed in Table 1. The FFPE tissue sections were subjected to IHC analyses. The most representative block of metastatic breast cancer tissue was selected after examination using H&E staining. The paraffin blocks were cut into 3‐μm‐thick tissue sections. Then, these sections were deparaffinized and rehydrated using xylene and alcohol solutions, respectively. IHC staining was performed using a Dako Autostainer Link 48 platform (Agilent Technologies, Santa Clara, CA, USA). Antigen retrieval was performed using EnVision FLEX Target Retrieval solution (1×; low pH) and EnVision FLEX wash buffer (1×). The staining process included the appropriate positive and negative controls.

**TABLE 1:** Clones, dilution, and source of the antibodies used.

Antibody	Company	Clone	Dilution
PD‐L1	Dako, Santa Clara, CA, USA	22C3	N/A
ER	Thermo Scientific, San Diego, CA, USA	SP1	1:100
PR	DAKO, Glostrup, Denmark	PgR	1:50
HER‐2	DAKO, Glostrup, Denmark	Polyclonal	1:1500
Ki‐67	Abcam, Cambridge, UK	SP6	1:100

### 2.3 Interpretation of Immunohistochemical Results

Nuclear staining values of 1% or higher were considered positive for ER (clone 6F11; dilution 1:200; Leica Biosystems, Wetzlar, Germany) and PR (clone 16; dilution 1:500; Leica Biosystems) [21]. HER2 (clone 4B5; dilution 1:5; Ventana Medical System, Oro Valley, AZ, USA) staining was performed according to the 2018 American Society of Clinical Oncology/College of American Pathologists [22]. Only samples with strong and circumferential membranous HER2 immunoreactivity (3+) were considered positive, whereas those with 0 or 1+ HER2 staining were considered negative. Cases with equivocal HER2 expression (2+) were further evaluated for HER2 gene amplification via silver in situ hybridization (SISH). Positive nuclear Ki67 (clone MIB; dilution 1:1000; Abcam, Cambridge, UK) staining was assessed based on the percentage of positive tumor cells, defined as the Ki67 labelling index (LI). The results of the PD‐L1‐specific IHC were interpreted considering the Tumor Proportion Score (TPS) and CPS. The TPS is calculated by dividing the number of PD‐L1‐positive tumor cells by the total number of viable tumor cells, multiplied by 100. A CPS of 10 or higher was considered positive [23]. CPS excluding PD‐L1 positive alveolar macrophages [CPS(NAM)] was defined as the CPS calculated by excluding the number of PD‐L1‐positive AMs from the number of PD‐L1‐positive cells. In this study, the calculation of CPS(NAM) was performed using the same paraffin block as that used for CPS evaluation. The paraffin block selected for evaluation was the representative block with the largest cross‐section of the tumor.

### 2.4 Tumor Phenotype Classification

In this study, breast cancer phenotypes were classified according to the IHC results for the ER, PR, HER‐2, and Ki‐67 LI statuses: *luminal A type*: ER and/or PR positive, HER‐2 negative, and Ki‐67 LI < 14%; *luminal B type*: (HER‐2 negative) ER and/or PR positive, HER‐2 negative, and Ki‐67 LI ≥ 14% and (HER‐2 positive) ER and/or PR positive and HER‐2 overexpressed and/or amplified; *HER‐2 type*: ER and PR negative and HER‐2 overexpressed and/or amplified; and *TNBC type*: ER, PR, and HER‐2 negative.

### 2.5 Statistical Analysis

Data were analyzed using SPSS for Windows (version 24.0; SPSS Inc., Chicago, IL, USA). Student's *t* test and Fisher's exact tests were used for continuous and categorical variables, respectively. Statistical significance was set at *p* < 0.05.

## 3 Results

### 3.1 Baseline Characteristics of Patients

A total 87 breast cancer patients with pulmonary metastasis were included in this study. The baseline characteristics of the patients are presented in Table 2. Among the study participants, 22 (25.3%) had luminal A breast cancer, 24 (27.6%) had luminal B breast cancer, 13 (14.9%) had HER‐2 positive breast cancer, and 28 (32.2%) had TNBC. The metastatic tumor size differed significantly across the subtypes (*p* = 0.031), with the largest tumor size (1.9 ± 2.0 cm) being observed for TNBC and the smallest (0.9 ± 0.3 cm) for HER‐2‐positive breast cancer.

**TABLE 2:** Clinicopathologic characteristics of lung metastasis patients according to the breast cancer phenotype.

Parameter	Total	Luminal A	Luminal B	HER‐2	TNBC^a^	*p*
	(*n* = 87)	(*n* = 22)	(*n* = 24)	(*n* = 13)	(*n* = 28)	
	(%)	(%)	(%)	(%)	(%)	
Age (years, mean ± SD)	52.9 ± 12.5	53.0 ± 12.1	54.9 ± 11.6	49.3 ± 11.6	52.8 ± 14.1	**0.640**
Tumor size (cm, mean ± SD)	1.4 ± 1.3	1.0 ± 0.5	1.3 ± 0.8	0.9 ± 0.3	1.9 ± 2.0	**0.031**
Estrogen receptor status						**< 0.001**
Negative	41 (47.1)	0 (0.0)	0 (0.0)	13 (100.0)	28 (100.0)	
Positive	46 (52.9)	22 (100.0)	24 (100.0)	0 (0.0)	0 (0.0)	
Progesterone receptor status						**< 0.001**
Negative	58 (66.7)	7 (31.8)	10 (41.7)	13 (100.0)	28 (100.0)	
Positive	29 (33.3)	15 (68.2)	14 (58.3)	0 (0.0)	0 (0.0)	
HER‐2 status						**< 0.001**
Negative	67 (77.0)	22 (100.0)	17 (70.8)	0 (0.0)	28 (100.0)	
Positive	20 (23.0)	0 (0.0)	7 (29.2)	13 (100.0)	0 (0.0)	
Ki‐67 LI (%)						**< 0.001**
≤ 14	26 (29.9)	22 (100.0)	1 (4.2)	0 (0.0)	3 (10.7)	
> 14	61 (70.1)	0 (0.0)	23 (95.8)	13 (100.0)	25 (89.3)	

*Note:* Bold indicates statistically significant value (*p* < 0.05).

^a^ TNBC, triple‐negative breast cancer.

### 3.2 Expression of Various PD‐L1 Clones in AMs in Lung Tissue

**FIGURE 1:**
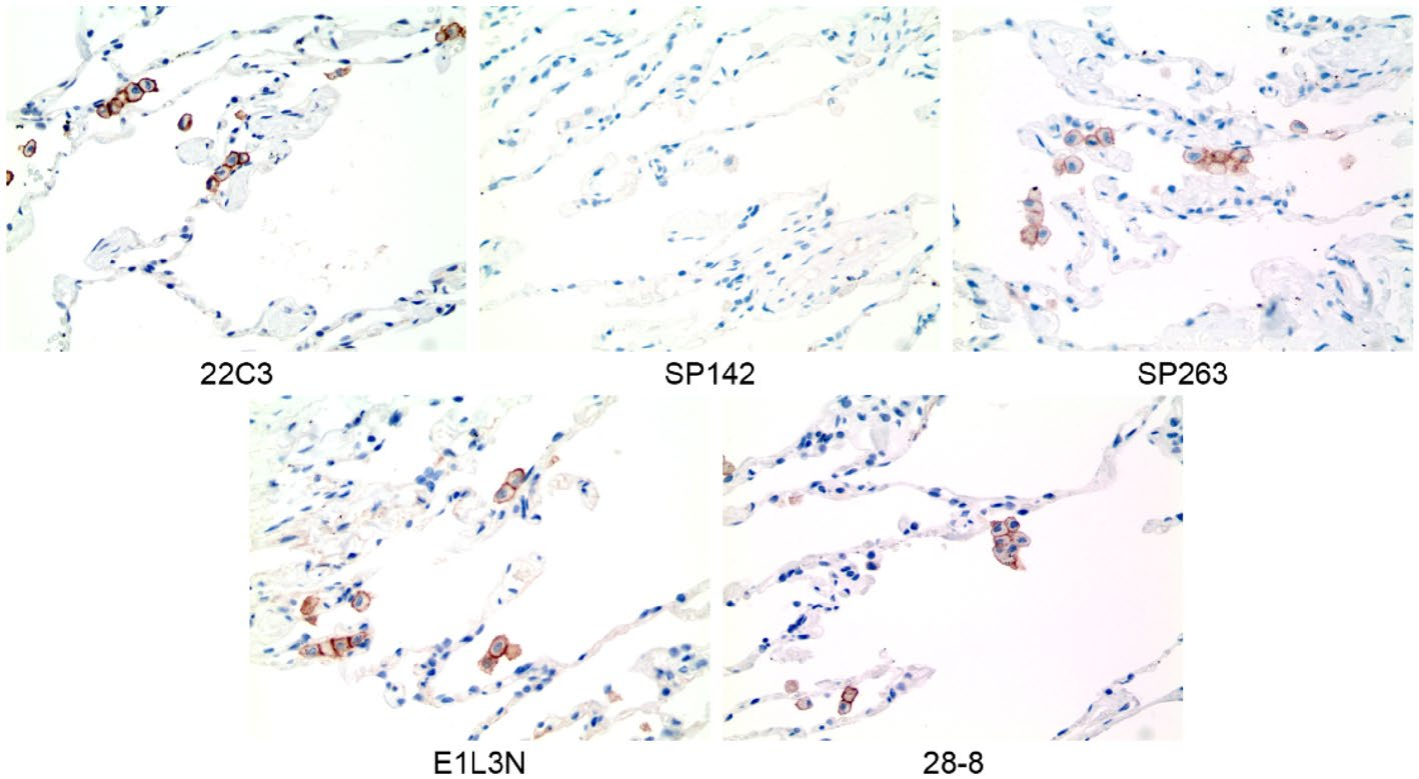
Expression of various PD‐L1 clones in alveolar macrophages in lung tissues. All clones (22C3, SP263, E1L3N, and 28–8) except SP142, PD‐L1 expression was detected along the membrane of alveolar macrophages. Magnification: 200×.

To determine the PD‐L1 expression in AMs present in normal human lung tissue, IHC was performed using antibodies against various PD‐L1 clones that are currently used in clinical practice (22C3, SP142, SP263, E1L3N, and 28–8). PD‐L1 expression specific to all clones except SP142 was observed along the membrane of AMs (Figure 1).

### 3.3 PD‐L1 Status in Lung Metastasis According to Breast Cancer Phenotype

The PD‐L1 status in lung metastasis according to the breast cancer phenotype is presented in Table 3. Patients were categorized for statistical analysis into three subgroups based on TPS (< 1, ≥ 1, and < 10, ≥ 10) and CPS (same subgroups), as well as CPS(NAM). There was a significant difference in TPS category distribution among the breast cancer phenotypes (*p* = 0.035), with the highest TPS (≥ 10) observed only in TNBC cases at 17.9%. Similarly, CPS category differences were significant (*p* < 0.001), with CPSs ≥ 1 and < 10 most common in HER‐2‐positive (46.2%) and TNBC (35.7%) cases. CPSs ≥ 10 were mainly found in luminal B (12.5%) and TNBC (35.7%) cases. The distribution of CPS(NAM) also varied significantly (*p* = 0.011), with CPS(NAM)s ≥ 1 and < 10 primarily in luminal B (8.3%) and TNBC (17.9%), and CPS(NAM)s ≥ 10 exclusively in TNBC cases (14.3%). A notable difference of ≥ 10 between CPSs and CPS(NAM)s was observed in five cases (5.7%): one in luminal B (4.2%) and four in TNBC (14.3%), as detailed in Table 4.

**TABLE 3:** TPS and CPS of PD‐L1 in lung metastasis according to the breast cancer phenotype.

Parameter	Total	Luminal A	Luminal B	HER‐2	TNBC^a^	*p*
	(*n* = 87)	(*n* = 22)	(*n* = 24)	(*n* = 13)	(*n* = 28)	
	(%)	(%)	(%)	(%)	(%)	
TPS						**0.035**
≥ 0, < 1	81 (93.1)	22 (100.0)	24 (100.0)	13 (100.0)	22 (78.6)	
≥ 1, < 10	1 (1.1)	0 (0.0)	0 (0.0)	0 (0.0)	1 (3.6)	
≥ 10	5 (5.7)	0 (0.0)	0 (0.0)	0 (0.0)	5 (17.9)	
CPS						**< 0.001**
≥ 0, < 1	51 (58.6)	20 (90.9)	16 (66.7)	7 (53.8)	8 (28.6)	
≥ 1, < 10	23 (26.4)	2 (9.1)	5 (20.8)	6 (46.2)	10 (35.7)	
≥ 10	13 (14.9)	0 (0.0)	3 (12.5)	0 (0.0)	10 (35.7)	
CPS						
Negative (CPS < 10)	74	22	21	13	18	
Positive (CPS ≥ 10)	13 (14.9)	0 (0.0)	3 (12.5)	0 (0.0)	10 (35.7)	
CPS(NAM)						**0.011**
≥ 0, < 1	76 (87.4)	22 (100.0)	22 (91.7)	13 (100.0)	19 (67.9)	
≥ 1, < 10	7 (8.0)	0 (0.0)	2 (8.3)	0 (0.0)	5 (17.9)	
≥ 10	4 (4.6)	0 (0.0)	0 (0.0)	0 (0.0)	4 (14.3)	
CPS(NAM)						
Negative (CPS < 10)	83	22	24	13	24	
Positive (CPS ≥ 10)	4 (4.6)	0 (0.0)	0 (0.0)	0 (0.0)	4 (14.3)	

*Note:* Bold indicates statistically significant value (*p* < 0.05).

^a^ TNBC, triple‐negative breast cancer.

**TABLE 4:** Difference between CPS and CPS(NAM) in lung metastasis according to the breast cancer phenotype.

Parameter	Total	Luminal A	Luminal B	HER‐2	TNBC^a^	*p*
	(*n* = 87)	(*n* = 22)	(*n* = 24)	(*n* = 13)	(*n* = 28)	
	(%)	(%)	(%)	(%)	(%)	
CPS‐CPS(NAM)						0.111
10 <	82 (94.3)	22 (100.0)	23 (95.8)	13 (100.0)	24 (85.7)	
10 ≥	5 (5.7)	0 (0.0)	1 (4.2)	0 (0.0)	4 (14.3)	

^a^ TNBC, triple‐negative breast cancer.

### 3.4 PD‐L1 Status Between CPS and CPS(NAM) in Lung Metastasis According to the Breast Cancer Phenotype

**FIGURE 2:**
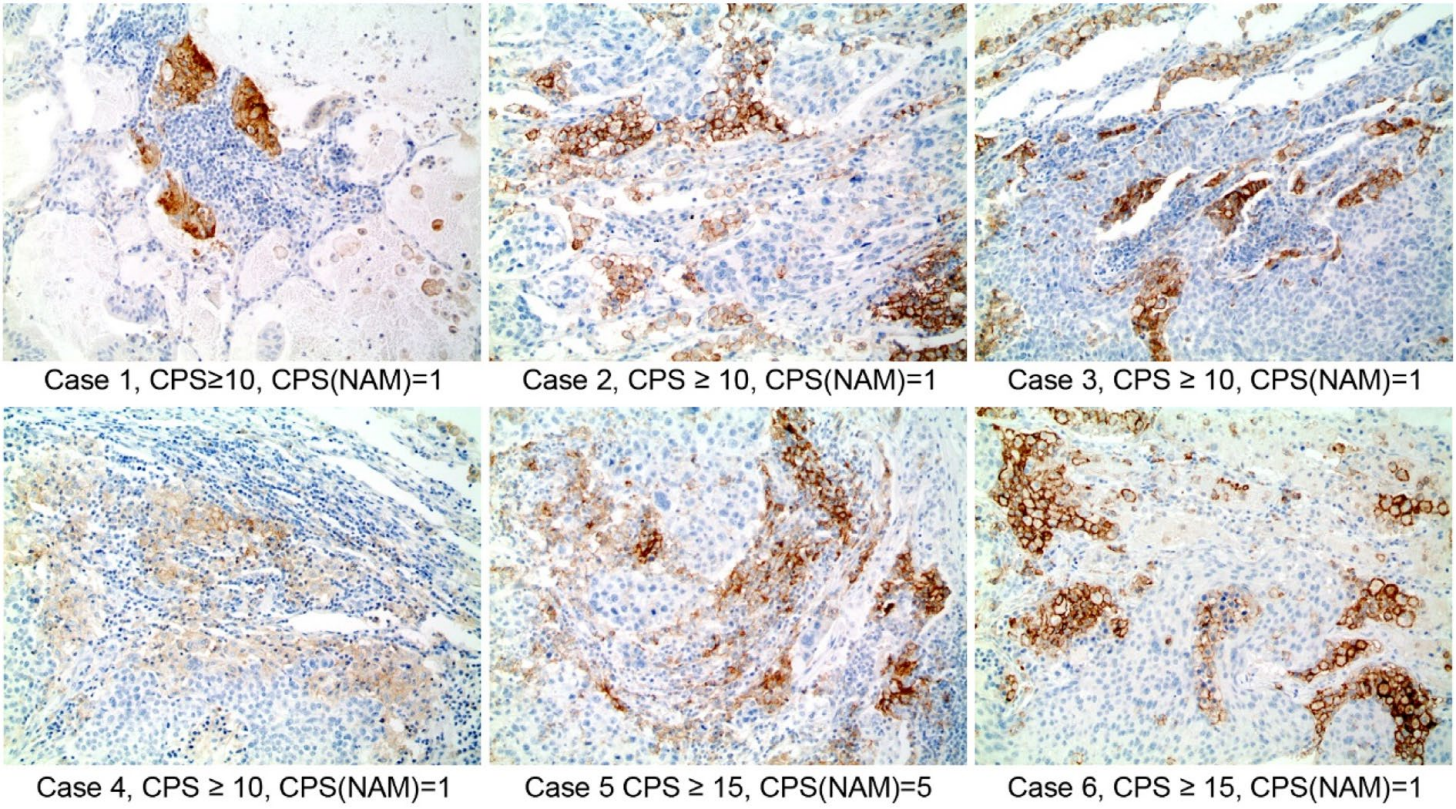
Conversion of PD‐L1 status between CPS and CPS(NAM) in breast cancer with pulmonary metastasis. From Case 1 to Case 4, PD‐L1 expression is predominantly observed in alveolar macrophages among the immune cells surrounding the tumor cells, yielding a CPS (combined positive score) of 10 or higher, indicative of PD‐L1 positivity. However, when considering CPS(NAM) in these cases, scores of ≤ 10 are recorded, suggesting PD‐L1 negativity. Both Case 5 and Case 6 exhibit a CPS of 15 or higher, suggesting PD‐L1 positivity. However, their CPS(NAM) scores, reflecting limited PD‐L1 expression in lymphocytes and excluding macrophages, fall to around 5 and 1 respectively, indicating PD‐L1 negativity.

**FIGURE 3:**
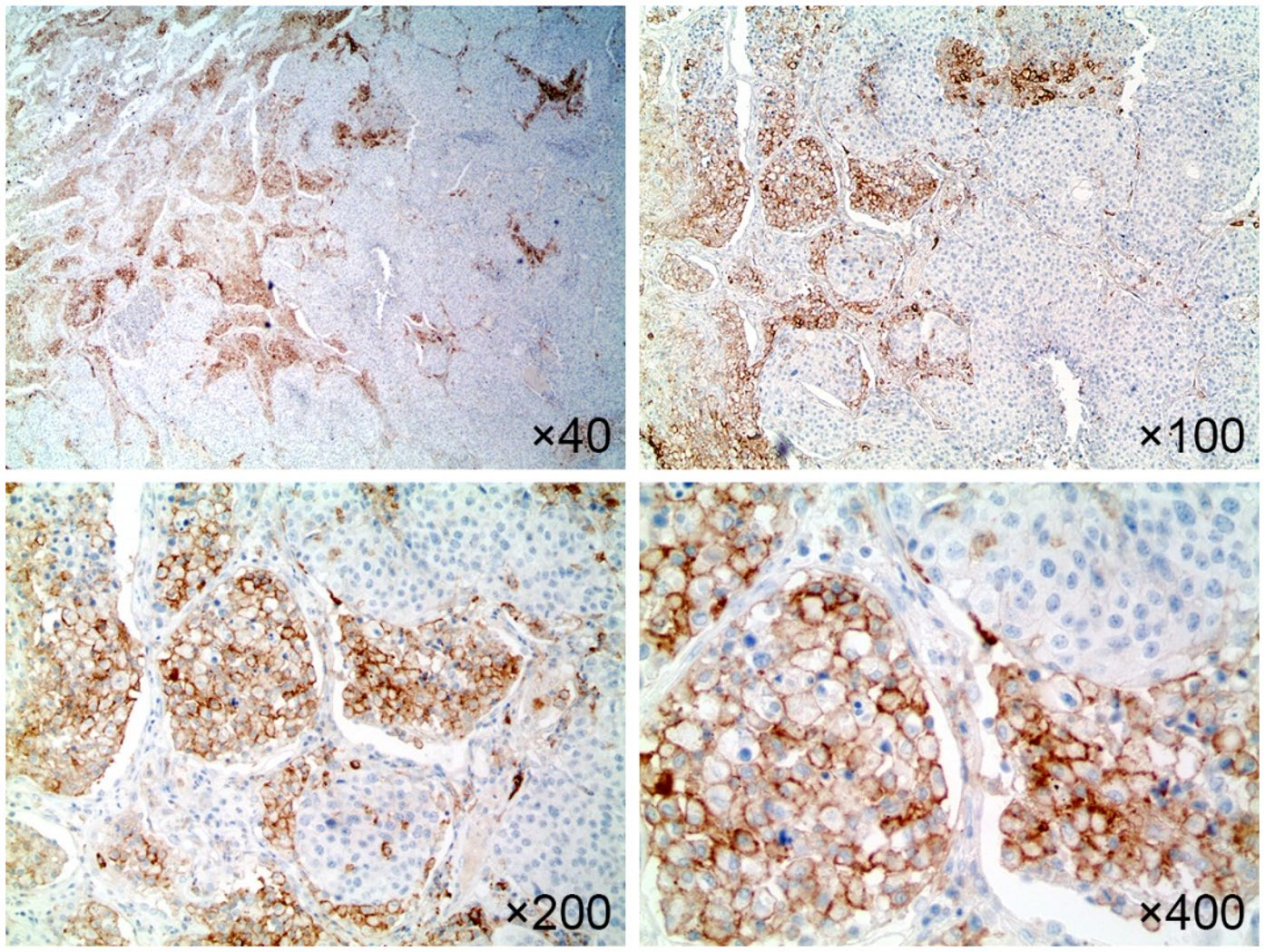
Representative case illustrating the conversion of PD‐L1 status between CPS and CPS(NAM) in breast cancer with pulmonary metastasis. At lower magnifications (40× and 100×), cells exhibiting strong PD‐L1 positivity are predominantly located at the tumor periphery, with some within the intratumoral area. At higher magnifications (200× and 400×), these PD‐L1‐expressing cells are identified as alveolar macrophages. Although the CPS for this case is sufficiently high (> 10), suggesting PD‐L1 positivity, the CPS(NAM) falls below 10, indicating PD‐L1 negativity.

When a CPS of 10 was used as the cutoff value for PD‐L1 (22C3), nine cases (10.3%) showed negative conversion when CPS(NAM) was considered (Figures 2 and 3). This change occurred in three cases of luminal B breast cancer (12.5%) and six cases of TNBC (21.4%), representing a significantly different prevalence among the breast cancer phenotypes (*p* = 0.007). This finding indicates a significantly higher negative conversion rate in TNBC (Table 5).

**TABLE 5:** PD‐L1 positive status between CPS and CPS(NAM) in lung metastasis according to the breast cancer phenotype.

Parameter	Total	Luminal A	Luminal B	HER‐2	TNBC^a^	*p*
	(*n* = 87)	(*n* = 22)	(*n* = 24)	(*n* = 13)	(*n* = 28)	
	(%)	(%)	(%)	(%)	(%)	
PD‐L1 positive status by CPS and CPS(NAM)						**0.007**
CPS and CPS(NAM) negative	74 (85.1)	22 (100.0)	21 (87.5)	13 (100.0)	18 (64.3)	
CPS and CPS(NAM) positive	4 (4.6)	0 (0.0)	0 (0.0)	0 (0.0)	4 (14.3)	
CPS positive→CPS(NAM) negative	9 (10.3)	0 (0.0)	3 (12.5)	0 (0.0)	6 (21.4)	

*Note:* Bold indicates statistically significant value (*p* < 0.05).

^a^ TNBC, triple‐negative breast cancer.

**FIGURE 4:**
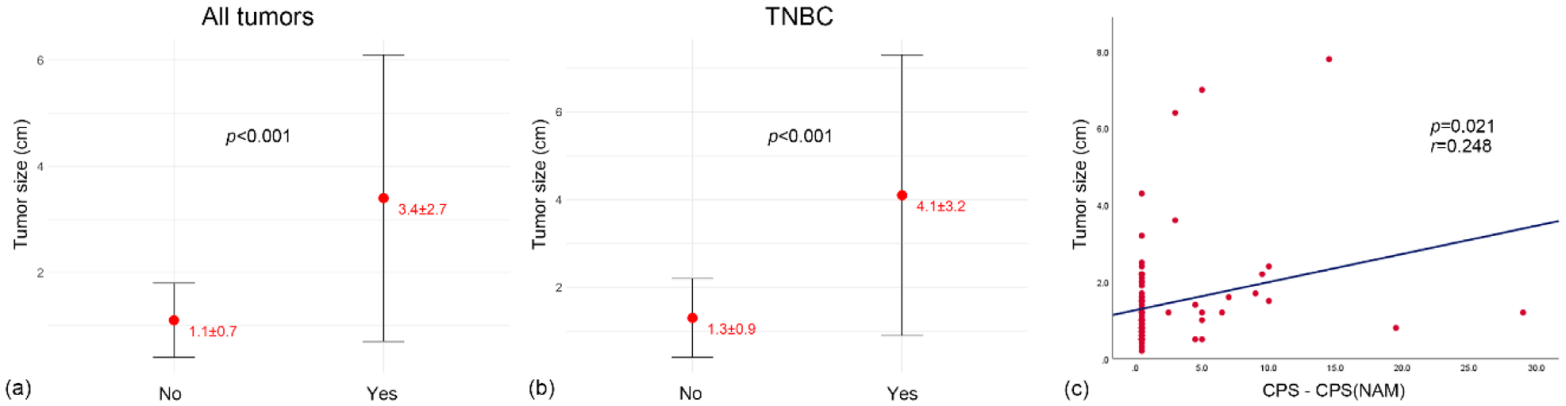
Correlation between metastatic tumor size and the difference between CPS and CPS(NAM). Metastatic tumor sizes were significantly larger in cases exhibiting a change from CPS‐positive to CPS(NAM)‐negative (Yes) compared to cases without this change (No), with significant differences noted (*p* < 0.001). (a) This pattern was consistent in TNBC tumors, with tumor sizes larger in CPS‐positive to CPS(NAM)‐negative conversions (*p* = 0.001). (b) Additionally, the difference between CPS and CPS(NAM) scores showed a positive correlation with metastatic tumor size (*p* = 0.021, r = 0.248). (c) Indicating that larger differences are associated with larger tumors.

The tumor size was significantly larger in cases with a CPS conversion result of CPS positive and CPS(NAM) negative (*p* < 0.001, Figure 4a); this difference remained significant in the TNBC‐specific analysis (*p* = 0.001, Figure 4b). Additionally, the difference between the CPSs and CPS(NAM)s showed a positive correlation with tumor size (*r* = 0.248, *p* = 0.021, Figure 4c).

## 4 Discussion

In this study, we evaluated the PD‐L1 (22C3) expression in tissue samples obtained from breast cancer patients with pulmonary metastasis and determined the impact of PD‐L1‐expressing AMs on the PD‐L1 status, measured as CPS. Initially, the rate of PD‐L1 (22C3) positivity (CPS ≥ 10) was 14.9% in all cases (35.7% in TNBC). Previous studies regarding TNBC reported PD‐L1 (22C3) positivity rates of 17% in primary tumors [24] and 66.7% in metastatic tumors [25]. Furthermore, it has been reported that the PD‐L1 positivity rate varies depending on the metastatic site in breast cancer, with bone showing the lowest rate (12%) and lymph nodes the highest (60%). The PD‐L1 expression rate in lung metastases is reported to be 52% (range: 43%–61%); however, direct comparisons between studies are limited due to differences in the antibody clones and scoring systems used [26]. The sites of metastasis in breast cancer, including the bone, liver, lungs, and brain, exhibit different metastatic patterns according to the molecular subtype [27]. In previous studies, TNBC accounted for the highest proportion of breast cancers with pulmonary metastasis (reportedly up to 33.1%), which is consistent with the data obtained in the present study (32.2%).

In the present study, cases with a CPS(NAM) ≥ 10 were consistently positive for PD‐L1, and all PD‐L1‐positive cases were TNBC cases (14.3%). Nine patients (10.3%) showed the negative conversion of CPS(NAM): 12.5% and 21.4% of cases with luminal B breast cancer and TNBC, respectively. PD‐L1 expression occurs both in tumor cells and in various immune cells present in the tumor microenvironment, which predominantly includes tumor‐associated macrophages (TAMs). Infiltration of TAMs significantly contributes to tumor invasion and metastasis [28, 29]. TAMs are typically classified into the M1 and M2 phenotypes, with M2 TAMs generally dominating and promoting tumor progression. The mechanism whereby M2 TAMs induce tumor progression involves the secretion of various cytokines and growth factors to stimulate tumor growth and invasion and angiogenesis, along with the suppression of T‐cell function, ultimately leading to immunosuppression [30, 31]. In breast cancer, progranulin increases PD‐L1 expression in TAMs via the JAK/STAT3 signaling pathway and promotes the M2 polarization of TAMs [32]. Theoretically, CPS(NAM) is not anticipated to exist in primary breast cancer, as CPS(NAM) originates from the presence of inherent PD‐L1‐positive alveolar macrophages, which are absent in breast tissue. In breast cancer, tumor‐associated macrophages or macrophages present in breast tissue typically do not exhibit inherent PD‐L1 positivity.

Two types of macrophages exist in the lungs: AMs and interstitial macrophages. AMs are primarily located on the luminal surface of lung tissue and are directly exposed to air and the surrounding environment, while interstitial macrophages are found in the lung tissue interstitium, particularly around the bronchi [33]. AMs originate hematopoietically from stem cells and constitute 60%–70% of the macrophages in the lung. Unlike the interstitial, peritoneal, and splenic macrophages, AMs have been reported to constitutively express PD‐L1 [20]. The inherent expression of PD‐L1 in AMs is suggested to be associated with their superior phagocytic ability and the inhibition of the activity of cytotoxic T lymphocytes, which in turn, contributes to optimal protective immunity and tolerance in the lungs. In the present study, AMs in human lung tissues inherently expressed PD‐L1 with all tested clones, except for SP142.

Pembrolizumab is currently approved by the FDA for patients with locally advanced or metastatic TNBC that yield positive results for the PD‐L1 22C3 PharmDx test, using a criterion of CPS ≥ 10. Therefore, when conducting the PD‐L1 22C3 PharmDx test on metastatic lung tissues from TNBC patients, it is important to consider that the level of AM infiltration could influence the CPS. The clinical significance and practical value of CPS(NAM) require further investigation. There are no studies that have proposed the concept of CPS(NAM) in breast cancer or other cancers, nor any research investigating whether CPS(NAM) is a more effective predictor of immune checkpoint inhibitor efficacy. If the therapeutic effects of immune checkpoint inhibitors in breast cancer lung metastases are found to be more influenced by CPS(NAM) than by CPS, this study would provide a strong rationale. This study was conducted on lung metastases of breast cancer tissues collected between 2005 and 2021. In South Korea, where this study was conducted, immunotherapy for advanced and metastatic TNBC based on PD‐L1 22C3 staining results was approved in July 2021. Consequently, most cases included in this study did not receive immunotherapy, making it challenging to evaluate responses to such treatments. Therefore, this hypothesis needs to be validated through future research and clinical trials. If the inherent PD‐L1 (22C3) expression in AMs does not affect tumor immunity or response to treatment, excluding the number of PD‐L1 positive AMs when evaluating the CPS should be actively considered.

In the present study, the fact that the transition from the CPS‐positive to the CPS(NAM)‐negative status occurred most frequently in TNBC (21.4%) underscores the heightened significance of including PD‐L1 positive AMs in CPS evaluation. The conversion from a positive CPS to a negative CPS(NAM) was significantly observed in larger tumors. This suggests that in TNBC lung metastasis, CPS is particularly influenced by AM infiltration, which positively correlates with tumor size.

AMs interact with tumor cells during the process of lung metastasis. In one proposed model, circulating progenitor cells of AMs are recruited to the tumor site by CCL2, which is secreted by interstitial macrophages. Subsequently, AMs contribute to tumor progression by secreting substances such as leukotriene B4 [34]. This model suggests a positive feedback loop between the progression of lung metastasis and AM recruitment. This aligns with our findings that larger tumor sizes are associated with increased AM infiltration in lung metastasis. Peritumoral AMs, which promote tumor growth by secreting IL‐10 and CCL2, have been implicated in poor prognosis in patients with lung metastases of non‐pulmonary origin and those with primary lung adenocarcinoma [35, 36]. Therefore, given the marked impact of peritumoral AMs on tumor biology, further research on the effects of inherent PD‐L1 22C3 expression in AMs on breast cancer with pulmonary metastasis is crucial.

This study has several limitations. First, the number of lung metastases from breast cancer included in this study was relatively small sample size leading to low statistical power. The small overall cohort size could further exacerbate these limitations in subgroup analyses. This limitation likely arises from our selection criteria, which restricted the inclusion to resected lung metastases. By analyzing whole tumor sections, we were able to assess the geometric distribution of AMs around the tumor and obtain more representative PD‐L1 expression data than would be possible with core needle biopsies or cytology specimens. Although we observed statistically significant differences among the tumor subtypes, a larger sample size would likely yield more reliable results. Based on the findings of this study, we anticipate further research involving multiple institutions in the future. Second, the clinical impact of the CPS in lung metastases, specifically regarding treatment response to immunotherapy and/or prognosis, was not evaluated in our study. Although pembrolizumab is approved by the FDA for use in treatment‐naive, locally advanced, or metastatic TNBC and has shown efficacy in a subset of high‐risk luminal tumors in clinical trials [37, 38], our study lacks data on these specific outcomes. Additionally, the PD‐L1 status in the primary breast tumor was not assessed. Studies indicate that CPS values tend to be higher in resection specimens compared to biopsy specimens of primary breast cancer [39], and discrepancies in PD‐L1 status based on the site of metastasis have been observed in breast cancer patients [26, 40]. However, data specifically concerning PD‐L1 status in lung metastases are relatively scarce. Our findings suggest that the lung may be a metastatic site particularly susceptible to increased CPS values due to AMs. Further studies involving precise comparative analyses of paired specimens from primary and metastatic sites are needed to guide optimal clinical assessments in patients with advanced breast cancer. In addition, a study investigating the difference in CPS between lung metastases and metastases in other organs in breast cancer patients with metastases to multiple organs, including the lungs, is an important and meaningful research direction that should be conducted in the future because no studies have investigated the differences in CPS between lung metastases and metastases in other organs. Third, as this study is a retrospective, single‐center study, it lacks an external validation cohort to verify the results. Last, as the study included all patients from a single institution who underwent surgery for breast cancer lung metastases during the study period and had paraffin blocks available in the pathology department, there is a potential risk of selection bias.

In conclusion, this study demonstrates that the inclusion or exclusion of AMs significantly influences CPS in lung metastases from breast cancer, particularly in TNBC. Additionally, larger tumor size plays a critical role in this evaluation. These findings highlight the importance of the tumor microenvironment in affecting CPS, which could impact therapeutic strategies in clinical practice.

## Author Contributions

All authors contributed to the study conception and design. Material preparation, data tabulation, and review were performed by Y.J.C. and J.S.K. (Immunohistochemistry interpretation); and Y.J.C. and H.M.K. (Data analysis). The first draft of the manuscript was written by Y.J.C. and J.S.K., and all authors commented on previous versions of the manuscript. All authors read and approved the final manuscript.

## Ethics Statement

This study was approved by the Institutional Review Board of the Severance Hospital (IRB number: 4–2024‐0276), specifically for the analysis of data collected between January 2005 and December 2021. The study conformed to the clinical practice guidelines of the Declaration of Helsinki (2013 amendment). Given its retrospective nature, informed consent was waived for all participants.

## Conflicts of Interest

The authors declare no conflicts of interest.

## Data Availability

The data presented in this study are available on request from the corresponding author.
